# Nasal and Ocular Immunization with Bacteriophage Virus-like Particle Vaccines Elicits Distinct Systemic and Mucosal Antibody Profiles

**DOI:** 10.3390/vaccines13080829

**Published:** 2025-08-03

**Authors:** Andzoa N. Jamus, Zoe E. R. Wilton, Samantha D. Armijo, Julian Flanagan, Isabella G. Romano, Susan B. Core, Kathryn M. Frietze

**Affiliations:** Department of Molecular Genetics and Microbiology, School of Medicine, University of New Mexico, Albuquerque, NM 87131, USA; anjamus@salud.unm.edu (A.N.J.); zwilton@salud.unm.edu (Z.E.R.W.); score@salud.unm.edu (S.B.C.)

**Keywords:** vaccine, antibody, intranasal, periocular, bacteriophage, virus-like particles

## Abstract

**Background/Objectives**: Intramuscular immunization elicits systemic IgG and is the primary route of vaccine administration in humans. However, there is growing interest in utilizing other routes of administration to tailor antibody profiles, increase immunity at primary sites of infection, simplify administration, and eliminate needle waste. Here, we investigated the antibody profiles elicited by immunization with bacteriophage virus-like particle vaccine platforms at various routes of administration. **Methods**: We chose two model bacteriophage vaccines for investigation: bacteriophage MS2 virus-like particles (VLPs) recombinantly displaying a short, conserved peptide from *Chlamydia trachomatis* major outer membrane protein (MS2) and bacteriophage Qβ VLPs displaying oxycodone through chemical conjugation (Qβ). We comprehensively characterized the antibodies elicited systemically and at various mucosal sites when the vaccines were administered intramuscularly, intranasally or periocularly with or without an intramuscular prime using various prime/boost schemes. **Results**: Intranasal and periocular immunization elicited robust mucosal and systemic IgA responses for both MS2 and Qβ. The intramuscular prime followed by intranasal or periocular boosts elicited broad antibody responses, and increased antibodies titers at certain anatomical sites. **Conclusions**: These findings demonstrate the tractability of bacteriophage VLP-based vaccines in generating specific antibody profiles based on the prime–boost regimen and route of administration.

## 1. Introduction

Vaccines are a cost-effective and effective strategy to control infectious diseases, benefiting overall public health. However, a vaccine’s ability to accomplish this depends on achieving sufficient protection to interrupt the target’s pathogenesis and transmission. In humans, vaccines are almost exclusively administered parenterally, namely intramuscularly and subcutaneously. This elicits a strong systemic immunoglobulin G (IgG) antibody response, but generally poor immunity at other mucosal sites [1]. Enhancing mucosal immunity may be beneficial, particularly for pathogens that infect at mucosal surfaces [2], including oral, anogenital, nasal, and ocular sites. This can be achieved by the addition of adjuvants that drive mucosal responses or administration of immunogens at mucosal sites [1].

Nasal immunization in particular can enhance immunoglobulin A (IgA) for various vaccine types [3,4], including those based on small RNA bacteriophage virus-like particles (VLPs) [5,6]. These bacteriophage VLPs are safe, highly immunogenic, self-assembling platforms comprising coat proteins that resemble the parent virus [7]. Two VLPs of note, derived from bacteriophages Qβ and MS2, have been investigated as immunogenic display platforms to develop vaccines against both infectious and non-infectious targets [8]. Both of these VLPs can stably display epitopes of interest, in a repeated fashion that elicits strong B-cell receptor crosslinking, and induces strong humoral immune responses [9]. Bacteriophage VLPs have a strong safety profile, both in preclinical [10,11] and clinical [12,13] studies. These bacteriophage VLPs have the potential to be “plug and play” vaccine platforms, and understanding general principles guiding their immunogenicity will help guide their effective use [14]. Additional research identifying ways to modify vaccine delivery to elicit a desired immune response against specific targets is needed.

Previously, we published studies investigating the use of bacteriophage VLPs as vaccines to elicit antibodies against oxycodone [15] (Qβ VLPs) and the variable domain 4 (VD4) of the Major Outer Membrane Protein of *Chlamydia trachomatis* (Ct), which functions as an adhesion factor [16] (MS2 VLPs). Both vaccine candidates were administered intramuscularly in initial studies and demonstrated promising efficacy in mouse models. Here, we use these vaccines (Qβ-OXY and MS2-VD4.A) as representative bacteriophage VLP vaccine models to investigate the impact of route of administration on the bacteriophage VLP-based vaccine-elicited antibody profile. We investigated systemic and mucosal IgG and IgA responses to vaccines administered via intramuscular, intranasal, and periocular routes with several different prime–boost strategies. Our data demonstrates the range of antibody profiles that can be elicited by bacteriophage VLP-based vaccines by modifying the route of administration. This work holds promise for expanding the utility of bacteriophage VLP-based vaccines, highlighting possible methods for bacteriophage VLP-based vaccine candidate optimization. 

## 2. Materials and Methods

### 2.1. Bacteriophage VLP Immunogen Production

Qβ VLPs were isolated and purified as previously described [15]. Briefly, the Qβ coat protein is expressed recombinantly in *E. coli* and self-assembled Qβ-VLPs are isolated and purified by size-exclusion chromatography. Qβ-VLPs are then stored at −20 °C until use. Display of oxycodone was accomplished by incubation with a bifunctional crosslinker, allowing the oxycodone-(Gly)_4_Cys hapten to be attached to surface-exposed lysine residues. Successful chemical conjugation was confirmed by SDS-PAGE and Coomassie blue staining.

MS2 VLPs were engineered and isolated as previously described [16]. Briefly, the coding sequence for MS2 coat protein single chain dimer was modified to include an insertion of the coding sequence for the VD4-MOMP conserved epitope. This plasmid was then used to recombinantly express the protein in *E. coli*. Self-assembling MS2-VD4.A VLPs were purified by size-exclusion chromatography and confirmed by SDS-PAGE prior to storage at −20 °C.

### 2.2. Vaccination

For intramuscular immunization, 5 μg of VLP was brought up to 50 μL of sterile PBS. This was injected with insulin needles in the hind leg muscle of BALB/c mice (male (n = 5) and female (n = 5)). For intranasal immunization, 5 μg of VLP was brought up to 15 μL of sterile PBS. This was then administered via drops from a P20 pipette until full volume was deposited in the nares. For periocular immunization, 5 μg of VLP was brought up to approximately 10 μL of sterile PBS. Mice were lightly anesthetize with inhaled isoflurane, and 5 μL drops were administered to each eye. Then, the eye lid was manually closed to retain the inoculum.

### 2.3. Sample Collection and Processing

To analyze systemic antibodies, retro-orbital bleeds were conducted 3 weeks after each dose (days 21, 42, and 63 post 1st immunization). Mice were put under isoflurane, and glass pipettes were used to prick the retro-orbital sinus and collect blood. Samples were then spun down twice to pellet red blood cells, and sera was collected in the supernatant.

To analyze ocular antibodies, lavages were performed at the same time points as previously described. Lavages were conducted 3 times in the collection week, allowing a day for recovery. Mice were put under isoflurane, and 10 μL of sterile PBS was dropped onto the eyelid, coating the surface. The PBS is then recollected. 10 μL coats and is recollected 3 times per eye, for a total 60 μL sample. As the collection occurred 3 times a week, they were then pooled together prior to analysis.

To analyze vaginal antibodies, lavages were performed at the same time points as previously described, as well as pooled after 3 collections in a week. Mice were put under isoflurane, and 30 μL of clean PBS was inserted intravaginally, resuspended, then recollected. 30 μL was inserted and recollected 3 times, for a total 90 μL per sample. Samples were spun down to pellet mucus, and supernatant was collected. As collection occurred 3 times a week, they were then pooled together prior to analysis.

To analyze anorectal antibodies, approximately 3–4 pellets were collected at time points previously described. Samples were snap frozen in −80 °C until processing. Fecal weights were recorded, and were suspended in 10% *w*/*v* of goat serum/PBS-T, similar to what has been previously performed [17]. Pellets were homogenized via vortex, then spun down, and the supernatant was collected prior to analysis.

### 2.4. ELISAs to Determine Antibody Titers

For MS2-VD4 antibodies, high-binding 96-well plates were coated with streptavidin, and incubated overnight at 4 °C or 2 h at 37 °C. After plates were washed 3 times with PBS, they were coated with SMPH and incubated for 1 h at room temperature with rocking. After plates were washed 3 times with PBS, plates were coated with linear CtSvDE peptide of VD4 MOMP protein, and incubated for 2 h at room temperature or overnight at 4 °C. Plates were washed 3 times with PBS, and blocked with 0.5% milk/PBS, incubated at room temperature for 1 h or overnight at 4 °C. Plates were washed 3 times with PBS, and samples that have been serially diluted were added to plates, and incubated for 2 h at room temperature with rocking. After plates were washed 5 times with PBS, secondary antibody (goat anti-mouse for IgG or IgA) in milk/PBS was added to plates at a 1:5000 dilution, and incubated for 45 min at room temperature with rocking. Plates were washed 3 times with PBS, and TMB was added to plates, incubated for 15 min (for sera IgG) or 25 min (for the rest of the samples and secondary antibodies). After incubation, 1% HCl was added to plates, and plates were read in plate reader at 450 nm.

For Qβ-OXY antibodies, high-binding 96-well plates were coated with OXY-BSA, and incubated overnight at 4 °C or 2 h at room temperature. After plates were washed 3 times with PBS, they were blocked with milk/PBS and incubated at room temperature for 1 h or overnight at 4 °C. The remainder of the ELISA was identical to those for MS2-VD4.A antibodies.

Samples were serially diluted down the plate to determine relative antibody titers, but were diluted differently, depending on the sample type. For sera IgG, sample was diluted starting at 1:40, and serially diluted 4-fold. For sera IgA, sample was diluted starting at 1:20, and serially diluted 2-fold. For ocular, vaginal, and fecal IgG and IgA, samples were diluted starting at 1:5, and serially diluted 2-fold.

### 2.5. Data Analysis of ELISAs

The end-point dilution of each sample was determined by the last dilution with absorbance at 450 nm higher than twice the average background of no serum control wells. Statistical analysis consisted of Kruskal–Wallis one-way ANOVA tests with multiple comparisons. Statistical analysis summary is found in Supplementary File S1, with *p* values listed.

## 3. Results

### 3.1. Experimental Design for Assessing Bacteriophage VLP Vaccine-Elicted Antibodies

**Figure 1:**
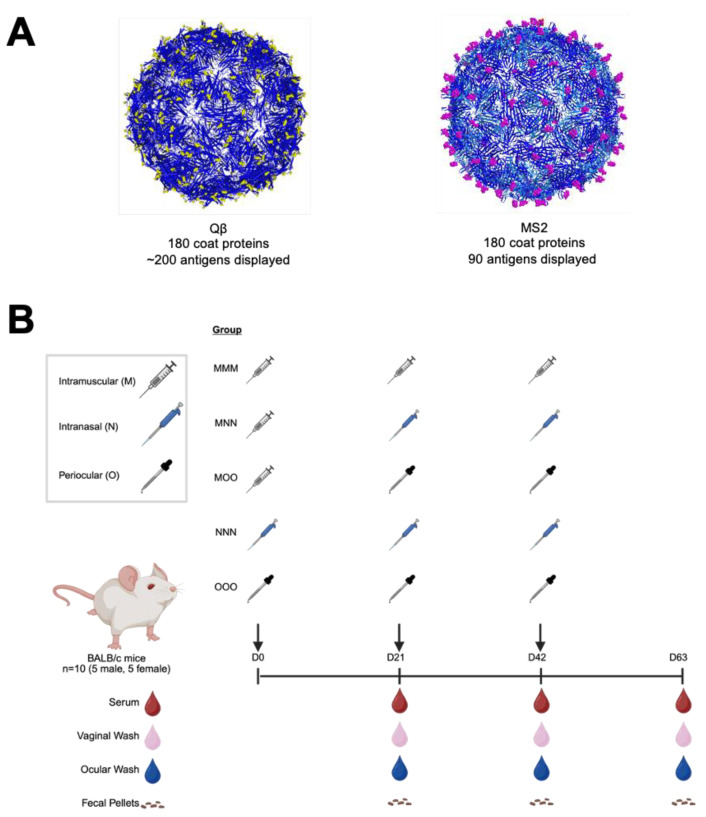
Experimental design for assessing routes of immunization with two model bacteriophage virus-like particle vaccine platforms. Bacteriophage VLP vaccines were generated with (**A**) MS2 recombinant display of a short *Chlamydia trachomatis* peptide (MS2) or Qβ chemically conjugated to oxycodone hapten (Qβ). These VLPs were then used to immunize male (n = 5) and female (n = 5) BALB/c mice via various routes of administration and boost schemes (**B**). Samples were collected at various times during the experiment and assessed for target-specific antibodies by ELISA.

In previous experiments, Qβ-OXY and MS2-VD4.A (Figure 1) showed promising protection against oxycodone and *Chlamydia*, respectively [15,16]. Here, we chose to investigate these two vaccines as models of bacteriophage VLP-based vaccines to examine the potential for optimizing antibody responses through altered routes of administration. To do this, we administered intranasal or periocular immunizations to mice, with or without a muscular prime dose, and compared the responses to those elicited by a standard 3-intramuscular-immunization scheme. Five vaccine groups of three doses each were included (Figure 1B). Male and female BALB/c mice were immunized with 5 μg of VLP in 50 μL (intramuscular), 15 μL (intranasal), or 10 μL (periocular) total volume in PBS. At various times post immunization, blood, vaginal wash, ocular wash, and fecal samples were collected and examined for target-specific (Oxycodone or Ct-VD4 peptide) antibody responses by ELISA. These VLPs will be referred to as Qβ and MS2 for simplicity. Groups will be referred to as MMM (all intramuscular), MNN (intramuscular prime, intranasal boosts), MOO (intramuscular prime, periocular boosts), NNN (intranasal only), and OOO (periocular only).

### 3.2. Intramuscular Immunization with Qβ and MS2 VLP-Based Vaccines Elicits High-Titer, Systemic IgG, Even with Subsequent Mucosal Boosts

**Figure 2:**
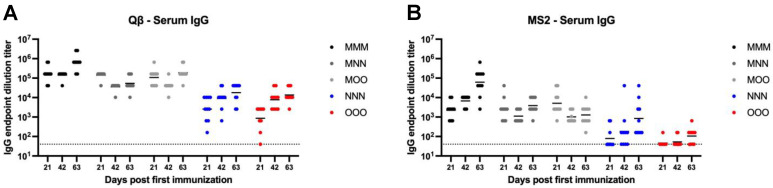
Serum IgG elicited after immunization. Mice (n = 10, 5 male, 5 female) were immunized as described in Figure 1 with (**A**) Qβ or (**B**) MS2, and serum was assessed for target-specific IgG end-point dilution titer by ELISA. Dotted line indicates lower limit of detection for the assay.

Systemic IgG is the most abundant antibody isotype, and it plays important roles in vaccine-mediated immunity. Because of this, it is important to understand the impact on systemic IgG when considering alternative routes of immunization. Qβ vaccination elicited >10^4^ mean endpoint dilution titer IgG in the serum for all immunization strategies by 63 days post first immunization, although this was achieved earlier for those groups that received an intramuscular prime (MMM, MNN, and MOO) (Figure 2A). Compared to MMM, NNN (Day 21—*p* = 0.0003, Day 42—*p* < 0.0001, Day 63—*p* < 0.0001) and OOO (Day 21—*p* < 0.0001, Day 42—*p* < 0.0001, Day 63—*p* < 0.0001) groups consistently showed significantly lower systemic IgG at all time points measured. This was also true for the MS2 vaccine (Figure 2B). Both NNN (Day 21—*p* = 0.001, Day 42—*p* < 0.0001, Day 63—*p* = 0.0014) and OOO (Day 21—*p* = 0.0026, Day 42—*p* < 0.0001, Day 63—*p* < 0.0001) groups had significantly lower IgG titers than the MMM group. Day 42 post first immunization, the MMM, MNN, and MOO groups had similar systemic IgG titers for both the Qβ and MS2 vaccine. This suggests while mucosal immunization can elicit detectable systemic IgG, muscular immunization elicits higher-titer systemic IgG.

### 3.3. Mucosal Immunization Alone or in Combination with Intramuscular Immunization Elicits Serum IgA

**Figure 3:**
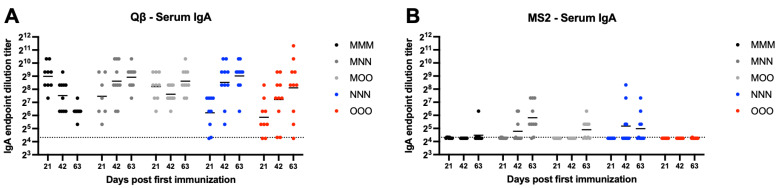
Serum IgA elicited after immunization. Mice (n = 10, 5 male, 5 female) were immunized as described in Figure 1 with (**A**) Qβ or (**B**) MS2, and serum was assessed for target-specific IgA end-point dilution titer by ELISA. Dotted line indicates lower limit of detection for the assay.

Systemic IgA is the second most abundant antibody isotype. Mucosal immunization elicited high-titer and sustained serum IgA compared to muscular only immunization. Qβ vaccination resulted in robust serum IgA by day 63 in all groups, although MMM serum IgA titers gradually decreased over time and all groups had statistically significantly higher serum IgA than MMM at day 63 (MNN—*p* = 0.0016, MOO—*p* = 0.0198, NNN—*p* = 0.0005, OOO—*p* = 0.0398) (Figure 3A). Many animals in the MS2 immunization groups did not generate detectable serum IgA responses, although detectable serum IgA was detected most frequently in groups that included a nasal immunization (MNN and NNN) (Figure 3B). These data suggest that a muscular prime is not critical in eliciting robust, sustained systemic IgA over time.

### 3.4. Vaginal IgG Is Robustly Elicited After Intramuscular or Mucosal Immunization

**Figure 4:**
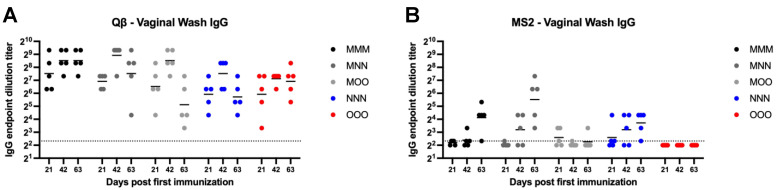
Vaginal wash IgG after immunization. Mice (n = 10, 5 male, 5 female) were immunized as described in Figure 1 with (**A**) Qβ or (**B**) MS2, and vaginal washes were assessed for target-specific IgG end-point dilution titer by ELISA. Dotted line indicates lower limit of detection for the assay.

We next examined vaginal IgG. Qβ vaccination groups generally were not statistically significantly different in vaginal wash IgG compared to MMM group (Figure 4A). However, MMM immunization resulted in more consistent, robust, and sustained vaginal IgG that showed sustained robust titers throughout the time course of the experiment, while responses in other groups showed more within-group variation. However, all animals in the Qβ experiment had detectable vaginal IgG for the duration of the experiment. For MS2, only the MMM, MNN and NNN groups elicited detectable vaginal IgG titers in all animals (Figure 4B). However, there were no statistically significant differences for any group in the experiment, except the OOO group which had significantly lower vaginal IgG than the MMM group at day 63 (*p* = 0.0246). No OOO animals vaccinated with MS2 had detectable vaginal wash IgG during the experiment.

### 3.5. Mucosal Immunizaiton Is Required for Sustained, Robust Vaginal IgA Responses

**Figure 5:**
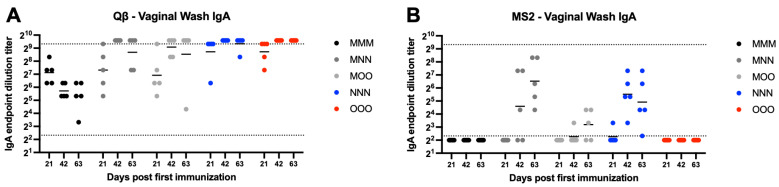
Vaginal wash IgA after immunization. Mice (n = 10, 5 male, 5 female) were immunized as described in Figure 1 with (**A**) Qβ or (**B**) MS2, and vaginal washes were assessed for target-specific IgA end-point dilution titer by ELISA. Dotted lines indicate lower and upper limit of detection for the assay.

For Qβ vaccinated animals, all mucosal immunization groups showed statistically significantly higher vaginal IgA compared to the muscular only MMM group (Figure 5A). By day 42, the majority of animals in the mucosal immunization groups (MNN, MOO, NNN, and OOO) were at or above the limit of detection for the assay. The MMM group had a sustained decrease in vaginal IgA over the course of the experiment ((MNN—*p* = 0.0010, MOO—*p* = 0.0393, NNN—*p* = 0.0010, and OOO—*p* = 0.0010). Similarly, the MMM group vaccinated with MS2 showed statistically significant lower vaginal IgA titers than the nasal immunization groups (MNN—*p* = 0.0024, NNN—*p* = 0.0277) (Figure 5B). Many animals vaccinated with MS2 did not show any detectable vaginal IgA during the experiment.

### 3.6. Variable Fecal IgG Is Elicited in Response to Bacteriophage VLP-Based Vaccine Administration

**Figure 6:**
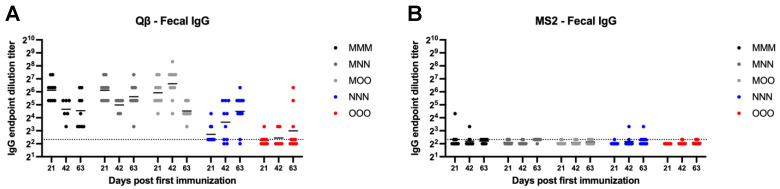
Fecal IgG after immunization. Mice (n = 10, 5 male, 5 female) were immunized as described in Figure 1 with (**A**) Qβ or (**B**) MS2, and fecal pellets were assessed for target-specific IgG end-point dilution titer by ELISA. Dotted line indicates lower limit of detection for the assay.

MS2 vaccinated animals generally showed very low or undetectable fecal IgG (Figure 6B). However, all Qβ animals vaccinated with a muscular prime (MMM, MNN, and MOO) showed detectable fecal IgG (Figure 6A). Rather high within-group variation was present in all groups which resulted in no statistically significant differences between groups for Qβ vaccinated animals, except for the statistically significant lower levels of fecal IgG in the NNN and OOO groups at day 21 (NNN—*p* = 0.015, OOO—*p* < 0.0001) (Figure 6A).

### 3.7. Fecal IgA Is Elicited After a Single Intramuscular Immunization and Is Sustained Regardless of Route of Administration for Subsequent Boosts

**Figure 7:**
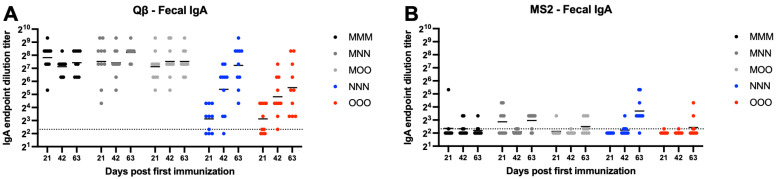
Fecal IgA after immunization. Mice (n = 10, 5 male, 5 female) were immunized as described in Figure 1 with (**A**) Qβ or (**B**) MS2, and fecal pellets were assessed for target-specific IgA end-point dilution titer by ELISA. Dotted line indicates lower limit of detection for the assay.

Qβ immunized animals that received a muscular prime (MMM, MNN, MOO) all generated detectable fecal IgA that was sustained for the duration of the experiment (Figure 7A). However, groups only receiving mucosal immunizations (NNN, OOO) had statistically less fecal IgA at day 21 (NNN—*p* < 0.0001, OOO—*p* = 0.0001). Over time, NNN animals generally experienced an increase in fecal IgA and this group was not statistically different than the MMM group at days 42 and 63. In contrast, although several OOO animals showed increased fecal IgA at days 42 and 63, this group remained statistically lower that MMM at days 42 (*p* = 0.0119) and 63 (*p* = 0.0143). Most MS2 vaccinated animals were at or below the limit of detection for fecal IgA, regardless of the vaccination scheme (Figure 7B).

### 3.8. Inconsistent Ocular IgG Is Elicited After Bacteriophage VLP-Based Vaccine Administration

**Figure 8:**
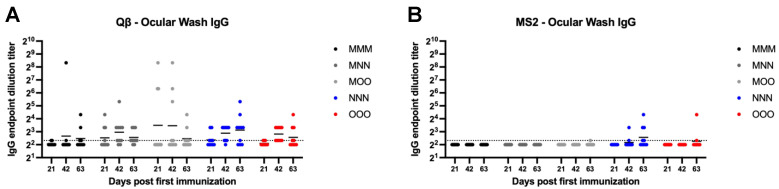
Ocular wash IgG elicited after immunization. Mice (n = 10, 5 male, 5 female) were immunized as described in Figure 1 with (**A**) Qβ or (**B**) MS2, and ocular washes were assessed for target-specific IgG end-point dilution titer by ELISA. Dotted line indicates lower limit of detection for the assay.

Ocular IgG was next examined using eye wash samples. Both MS2 and Qβ vaccination inconsistently elicited ocular IgG (Figure 8). In all Qβ groups examined at least several animals showed detectable ocular IgG by the day 63 (Figure 8A). However, only MOO, NNN, and OOO groups showed any animals with detectable ocular IgG after vaccination with MS2 (Figure 8B).

### 3.9. Mucosal Administration Is Required to Elicit Robust and Sustained Ocular IgA After Bacteriophage VLP-Based Vaccination

**Figure 9:**
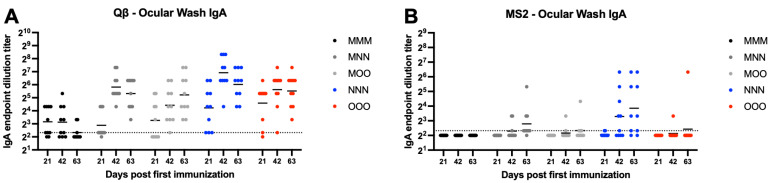
Ocular wash IgA after immunization. Mice (n = 10, 5 male, 5 female) were immunized as described in Figure 1 with (**A**) Qβ or (**B**) MS2, and ocular washes were assessed for target-specific IgA end-point dilution titer by ELISA. Dotted line indicates lower limit of detection for the assay.

Ocular IgA was assessed next using ELISA to detect IgA in ocular wash samples. For Qβ, all groups except MMM had all animals that exhibit detectable ocular IgA by day 63 (Figure 9A). All groups showed statistically significant increases in ocular IgA compared to MMM at day 63 (MMN—*p* = 0.0024, MOO—*p* = 0.0034, NNN—*p* < 0.0001, OOO—*p* = 0.0008). For MS2, the majority of animals from all groups showed undetectable or very low ocular wash IgA, except the NNN group at day 63 (*p* = 0.0005) (Figure 9B).

## 4. Discussion

Here we describe an extensive assessment of antibody profiles elicited in response to vaccination at different anatomical sites using two bacteriophage VLP-based vaccines as models. Overall, we observed improved mucosal site IgA antibody titers when vaccine was administered at mucosal sites as either a prime or boost dose. While MNN and MOO were generally equivalent to MMM in eliciting serum, vaginal, fecal and ocular IgG, MNN and MOO were superior to MMM at eliciting IgA in all samples except fecal. These data support the potential for bacteriophage VLP-based vaccines to be tailored to elicit specific IgG and IgA profiles through route of administration prime/boost strategies. A summary of our findings can be found in Supplementary Figure S1.

One interesting finding from our investigation was the ability of periocular boost to elicit IgA at comparable levels as intranasal boost. For almost all samples assessed, MOO and MNN elicited IgG and IgA titers. Although nasal boost elicited higher titers in some samples. This opens opportunities to explore ocular delivery of bacteriophage VLP-based vaccines. Periocular immunization, or “eyedrop vaccines” may be beneficial in resource poor areas when skilled health workers are not available to administer vaccines. Additionally, there is interest in utilizing “eyedrop vaccines” to increase immunity against pathogens that can cause damage to the eye [18]. Although we did not observe vaccination site irritation upon periocular immunization, this was not a planned endpoint, so additional studies are needed to examine additional safety parameters. Eyedrop delivery of ocular medications is generally well-accepted by patients as there are many over-the counter eyedrop available, indicating eyedrop delivery could be a useful option to expand self-delivery of vaccines.

While periocular immunization elicited high titer antibodies in many of the samples tested, overall nasal immunization seemed to have higher immunogenicity. This may be due to the immune processing of the vaccine via mucosa-associated lymphoid tissues (MALTs). VLPs administered via muscular immunization drain to lymph nodes either themselves or via uptake from antigen-presenting cells, depending on the VLP [14]. VLPs via mucosal immunization drain to local naïve B and T cells in the MALTs. These tissues are located in areas including the eye (conjunctiva-associated), nose (nasal-associated), the GI tract (gut-associated), and vaginal tract (vulvo-vaginal-associated). B and T cells will mature at these MALTs and then travel to lymphoid tissues at other mucosal sites [19]. Nasal immunization compared to ocular immunization causes drainage to different MALTs, which may impact vaccine immunogenicity and possibly efficacy. This may be due to differences in the population sizes of naïve lymphocytes present to interact with the vaccine near the eye compared to the nose. As the eye is immune privileged, a decreased immune presence might be limiting the response to vaccination, but further investigation is needed.

While there were overall similar shifts in antibody profiles shared between the two bacteriophage VLP vaccines, we saw overall lower endpoint dilution titers against the target antigen for MS2. This could be due to differences in the number of antigen display sites between the two VLPs. The Qβ VLP has three to four surface-exposed lysines on each coat protein that function as are attachment sites for antigens of interest. Our previous work showed that the Qβ-OXY vaccine displays approximately 200 oxycodone molecules per VLP [15]. This differs greatly from the MS2 VLP, which is comprising 90 single-chain dimers that each display one epitope of interest on the AB loop [8]. Fewer display sites on MS2 may yield a more muted immune response compared to Qβ. Although we used two VLP-based vaccines here as models of small RNA bacteriophage VLP-based vaccines, it is impossible to correlate these differences in antibody titers to one VLP vaccine platform being more efficacious. The selection of a VLP platform is highly dependent on many factors, such as antigen size and structural display.

Enhancing mucosal immunity through vaccination may be beneficial, particularly for pathogens that enter at mucosal surfaces. Currently, there is a lack of mechanistic understanding that could be helpful in guiding best practices in VLP-based vaccine design. Qβ VLPs in particular have been investigated as a universal vaccine platform for a variety of pathogens and other antigens. Expanding the opportunities for this and other bacteriophage VLP-based vaccines to target mucosal pathogens by tailoring mucosal antibody profiles through routes of administration could further enhance this “plug and play” vaccine platform.

Previous work with other VLPs have shown that intranasal immunization induces both systemic and mucosal IgG and IgA, while parenteral immunization only elicits systemic and mucosal IgG [5,6]. Additional research is needed in identifying ways to modify these vaccines to tailor their use against specific pathogens. The route of immunization and prime–boost scheme may influence vaccine efficacy and make the difference in eliciting protection. This may be true for the specific vaccines that we used in this report (Qβ-OXY and MS2-VD4.A). Recent data suggests that vaccine-elicited systemic IgA is correlated with protection from opioids [20,21], and eliciting mucosal IgG and IgA may enhance protection against *Chlamydia trachomatis*, which infects at a variety of mucosal sites.

Overall, these findings further characterize strategies to elicit systemic and mucosal immunity in VLP-based vaccines. In situations where a broad response would be beneficial, such as for mucosal pathogens, adding a mucosal boost after an intramuscular immunization could help provide additional protection. A prime–boost strategy utilizing a muscular prime immunization followed by two mucosal immunization elicited high-titer IgA and IgG titers both systemically and at several different anatomical sites. Mucosal VLP administration alone can elicit high titer antibodies, especially serum IgA and antibody titers at mucosal sites without the need for adjuvant. Mucosal administration is beneficial as it reduces the need for trained personnel and the production of needle waste. The production of self-administered nasal spray or eyedrop vaccines could be advantageous in resource-poor or remote areas that may lack trained medical personnel. Additionally, individuals with a phobia of needles may benefit from these alternative routes of administration.

## 5. Conclusions

These findings demonstrate that antibody profiles elicited by bacteriophage VLP-based vaccines can be altered by the modification of the route of administration and prime–boost regimen. Mucosal administration, particularly after an intramuscular prime, elicited both IgG and IgA at all mucosal sites tested. This work holds promise for expanding the utility of these types of vaccines, elucidating methods that can further optimize various candidates. Future work includes performing similar investigations in more bacteriophage VLP-based vaccines by targeting different antigens and utilizing different VLP platforms. Further understanding this phenomenon across VLP-based vaccines is critical to inform future vaccine development efforts, as both the VLP platform and the route of administration can alter the immune response.

## Data Availability

Data are either presented in this study or are available upon request by e-mailing the corresponding author.

## Supplementary Materials

The following supporting information can be downloaded at: https://www.mdpi.com/article/10.3390/vaccines13080829/s1, File S1: Statistical analysis summary, Figure S1: A summary of findings.
