# Supplementary material for: Nasal and Ocular Immunization with Bacteriophage Virus-like Particle Vaccines Elicits Distinct Systemic and Mucosal Antibody Profiles
Source: Vaccines (Basel). 2025 Aug 3;13(8):829. doi: 10.3390/vaccines13080829 (PMC12389891; doi:10.3390/vaccines13080829)
Supplement: Supplementary file 1 [file vaccines-13-00829-s001.zip › Supplemental Figure S1.pdf]

A

Qbeta Immunization

	MNN	MOO	NNN	OOO
**Systemic IgG**	Decreased	NS	Decreased	Decreased
**Systemic IgA**	Increased	Increased	Increased	Increased
**Vaginal Wash IgG**	NS	Decreased	Decreased	NS
**Vaginal Wash IgA**	NS	Increased	Increased	Increased
**Fecal IgG**	NS	NS	NS	NS
**Fecal IgA**	NS	NS	NS	Decreased
**Eyewash IgG**	NS	NS	NS	NS
**Eyewash IgA**	Increased	Increased	Increased	Increased

B

MS2 Immunization

	MNN	MOO	NNN	OOO
**Systemic IgG**	NS	Decreased	Decreased	Decreased
**Systemic IgA**	Increased	NS	NS	NS
**Vaginal Wash IgG**	NS	NS	NS	Decreased
**Vaginal Wash IgA**	Increased	NS	Increased	NS
**Fecal IgG**	NS	NS	NS	NS
**Fecal IgA**	NS	NS	Increased	NS
**Eyewash IgG**	NS	NS	Increased	NS
**Eyewash IgA**	Increased	NS	Increased	NS

Supplemental Figure S1: Comparison of antibody titers at day 63 for the Qβ (**A**) or MS2 VLPs (**B**). The antibody titers at different anatomical sites were compared between the MMM group and the other immunization groups. Statistically significant changes in the antibody titers were reported (based on one-way ANOVA analysis: NS = not significant, Decreased = significantly decreased antibody titer compared to the MMM group, Increased = significantly increased antibody titer compared to the MMM group).
